# Improving Guava Shelf Life and Preserving Postharvest Quality With Edible Coatings

**DOI:** 10.1002/fsn3.70491

**Published:** 2025-06-20

**Authors:** Litun Ahmed Labib, Swagata Ahmed, Md. Fakhrul Hasan

**Affiliations:** ^1^ Department of Horticulture Patuakhali Science and Technology University Patuakhali Bangladesh; ^2^ Department of Horticulture Bangabandhu Sheikh Mujibur Rahman Agricultural University Gazipur Bangladesh

**Keywords:** *Aloe vera* gel, chitosan, cinnamon essential oil, edible coating, food quality, guava ( *Psidium guajava* L.), gum arabic, postharvest operation, propolis, shelf life extension

## Abstract

Guava ( *Psidium guajava* L.) is a nutrient‐dense climacteric fruit, but is prone to rapid postharvest deterioration due to physicochemical changes, leading to substantial quality and economic losses. This study investigated the efficacy of edible coatings—chitosan 2% (CH 2%), cinnamon essential oil 2% (CEO 2%), *aloe vera* gel 30% (AVG 30%), propolis 10% (PR 10%), and gum arabic 10% (GA 10%)–applied individually and in combination (15 total formulations, excluding control) to extend shelf life and maintain postharvest quality. Fruits treated with these coatings were stored under ambient conditions (20°C ± 1°C) and compared to uncoated controls. Results revealed that edible coatings significantly (*p* ≤ 0.05) delayed deteriorative processes, with the CH 2% + GA 10% blend demonstrating optimal performance. Notably, this formulation minimized weight loss (6.31%), retained firmness (4.11 kg/cm^2^), and preserved color attributes. Additionally, it maintained superior biochemical quality, including titratable acidity (0.322%), ascorbic acid (198.22 mg/100 g), total antioxidants (136.76 mM Trolox/100 g), and phenolic content (177.03 mg GAE/100 g), with extended shelf life up to 12 days. The findings underscore the potential of chitosan‐gum arabic composite coatings as a sustainable, natural solution to mitigate postharvest losses in guava, offering actionable strategies for enhancing storability and marketability without reliance on synthetic additives.

## Graphical abstract

Guava ( *Psidium guajava* L.) is a climacteric fruit prone to rapid spoilage due to its delicate skin. This study investigated the impact of various edible coatings on the shelf life and physicochemical properties of guava. The results demonstrated that coatings significantly (*p* ≤ 0.05) extended the shelf life and preserved its nutritional integrity.

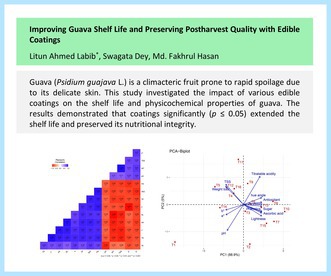

**Abbreviations**
a*
: redness
AVG:
*aloe vera* gel
b*
: yellowness
CEO: cinnamon essential oil
CH: chitosan
DS: days of storage
GA: gum arabic
*h°*
: hue angle
L*
: lightness
PR: propolis
TA: titratable acidity
TPC: total phenolic content
TSS: total soluble solids

## 1 Introduction

Guava ( *Psidium guajava* L.) is a widely cultivated fruit in subtropical and tropical regions, renowned for its delicious taste and high nutritional value. It is an excellent source of antioxidants, soluble sugars, proteins, dietary fiber, riboflavin, essential amino acids, and contains up to four times more vitamin C than a typical orange (Khan et al. 2025; Mathiazhagan et al. 2023). Additionally, guava contains a variety of bioactive compounds, including flavonoids, tannins, carotenoids, polyphenols, and pentacyclic triterpenoids (Hussain et al. 2021; Vijaya Anand et al. 2020). These compounds contribute to a range of health benefits, including antimicrobial, antidiarrheal, antidiabetic, anticancer, anti‐inflammatory, immune‐modulatory, and cardioprotective effects (Jamieson et al. 2022; Naseer et al. 2018; Upadhyay et al. 2019). In Bangladesh, guava holds significant economic importance, with an annual production of approximately 256,105.56 metric tons from 45,386.94 acres of land (BBS 2024).

Despite the enormous health benefits and high market demand, the thin and delicate skin of guava presents considerable challenges for the industry. The skin is highly susceptible to damage, dehydration, and deterioration, resulting in a notably short postharvest shelf life of just 3–4 days at room temperature (25°C ± 2°C) (Francisco et al. 2020; Yousaf et al. 2024). As a climacteric fruit, guava undergoes rapid physiological changes after harvest, exhibiting a high respiratory rate and swift maturation when stored under ambient conditions (Gull et al. 2024). This elevated respiration is governed by ethylene, a natural plant hormone produced through a complex signaling pathway involving L‐methionine and the enzyme 1‐aminocyclopropane‐1‐carboxylic acid (ACC) synthase (Tipu and Sherif 2024). As a result of these metabolic shifts, guava fruits experience accelerated senescence, leading to significant deterioration, such as weight loss, reduced nutritional content, loss of turgidity, chlorophyll degradation, and ultimately diminished marketability (Feng et al. 2021; Shanta et al. 2023; Zhang 2024). In Bangladesh, approximately 30%–40% of guava is wasted due to its high perishability and short shelf life (Dutta Roy et al. 2023). This has created a pressing need for the development of innovative technologies to enhance its shelf life.

Traditional methods using synthetic additives like formaldehyde, BHA, and BHT are becoming less popular due to health and safety concerns among consumers (Nur Hanani et al. 2023). As alternatives, techniques such as UV‐C, non‐thermal methods like ultrasound and ozone, and thermal treatments have been explored to control microbial growth and maintain fruit quality (Guevara et al. 2012; Noguera et al. 2021). However, these methods often require specialized equipment, which may not be affordable to small‐scale producers. In contrast, edible coatings have emerged as a promising, sustainable alternative to chemical preservatives (Chen et al. 2019; Yan et al. 2019). A thin, transparent layer applied directly to the fruit's surface can enhance shelf life by reducing weight loss, respiration rates, oxidative damage, and physiological disorders (Hasan et al. 2022; Formiga et al. 2022; Ali et al. 2025). Additionally, they can deliver natural additives that preserve freshness and enhance the fruit's appearance (Kohli et al. 2024; Kaur et al. 2024a; Lo'ay and El‐Khateeb 2018). Edible coatings provide a cost‐effective and eco‐friendly way to preserve food, benefiting both consumer health and the environment (Kaur, Somasundram, Razali, Mourad, et al. 2024).

Edible coatings derived from natural biomaterials, including polysaccharides, proteins, and lipids, have emerged as promising solutions for enhancing the shelf life and maintaining the nutritional integrity of various fruits (Chen et al. 2019; Yan et al. 2019; Hasan et al. 2022). Among the widely studied natural biomaterials, Chitosan (CH), Cinnamon essential oil (CEO), *Aloe vera* gel (AVG), Propolis (PR), and Gum arabic (GA) stand out for their exceptional preservative properties, which have garnered significant attention in recent years (Eshetu et al. 2019; Yu et al. 2021; Dutta Roy et al. 2023; Segueni et al. 2023; Tiamiyu et al. 2023; Ahmed et al. 2024; Kaur et al. 2024b). CH, derived from the deacetylation of chitin, is a versatile biopolymer with excellent film‐forming ability, biocompatibility, and antimicrobial properties (Agarwal et al. 2021; Asif et al. 2023). CEO, rich in aldehydes, is known for its potent antimicrobial and antioxidant effects and plays a vital role in food preservation (Liu et al. 2021). AVG, rich in starch and with antimicrobial properties, helps prevent moisture loss while retaining fruit firmness (Ahmed et al. 2024). PR, abundant in bioactive compounds, offers significant antimicrobial, antifungal, and antioxidant benefits, extending the shelf life of food products (Segueni et al. 2023). GA, a natural emulsifier and film‐forming agent, is widely used for its ability to delay the physicochemical alterations of food (Tiamiyu et al. 2023).

However, limited research exists on the effectiveness of edible coatings in preserving the shelf life and nutritional qualities of guava. Identifying an ideal edible coating could provide a crucial solution for reducing postharvest losses and improving the storage quality of guava. Therefore, the main objective of this study was to develop different edible coatings using CH, CEO, AVG, PR, and GA, and evaluate their impacts on the shelf life and physicochemical properties of guava during storage.

## 2 Materials and Methods

### 2.1 Materials

Fresh, commercially mature, uniform, and disease free ‘Thai 5’ (commercial variety) guava fruits were collected from Swarupkathi, Barisal, Bangladesh (22.74496°N, 90.11674°E). The physicochemical properties of the fresh guava are presented in Table 1. CH was procured from Spectrum Chemical Mfg. Corp., USA. Food‐grade CEO from *Cinnamomum zeylanicum* bark was supplied by Zardband Pharmaceuticals, Iran. Fresh, disease‐free *aloe vera* leaves were collected from the Horticulture Germplasm Centre, Patuakhali Science and Technology University (PSTU), Bangladesh. Crude PR was obtained from the Bangladesh Institute of Apiculture (BIA) and stored in aluminum foil at 10°C in dark conditions until extraction. Food‐grade GA powder was purchased from Merck Life Science, Germany. All chemicals used in the experiment were of analytical grade. Upon arrival, guava fruits and *aloe vera* leaves were thoroughly cleaned, first by washing and then disinfected with 0.01% NaOCl for 2 min. After disinfection, both were rinsed with distilled water and air dried at 25°C ± 2°C for 90 min before further processing.

**TABLE 1:** Physicochemical properties of fresh guava.

Parameters	Values
Weight (g)	217.33 ± 8.02
Moisture (%)	84.56 ± 0.26
Firmness (kg/cm^2^)	4.68 ± 0.10
Lightness (L^*^)	71.89 ± 0.10
a^*^	−10.25 ± 0.08
b^*^	29.73 ± 0.07
Hue angle (*h°*)	109.02 ± 0.18
Total soluble solids (% Brix)	7.78 ± 0.09
pH	3.96 ± 0.05
Titratable acidity (%)	0.384 ± 0.01
Total sugar (%)	9.81 ± 0.23
Ascorbic acid (mg/100 g)	212.63 ± 0.34
Total phenolics (mg GAE/100 g)	208.29 ± 0.41
Antioxidant (mM Trolox/100 g)	138.14 ± 0.17

*Note:* * All values are presented as mean ± SD.

### 2.2 Preparation and Application of Edible Coatings

Sixteen different treatments were prepared as follows: T1—Control (uncoated), T2—CH 2%, T3—CEO 2%, T4—AVG 30%, T5—PR 10%, T6—GA 10%, T7—CH 2% + CEO 2%, T8—CH 2% + AVG 30%, T9—CH 2% + PR 10%, T10—CH 2% + GA 10%, T11—CEO 2% + AVG 30%, T12—CEO 2% + PR 10%, T13—CEO 2% + GA 10%, T14—AVG 30% + PR 10%, T15—AVG 30% + GA 10%, and T16—PR 10% + GA 10%. CH coatings were prepared according to Xing et al. (2011) with minor modifications, where 2% CH (w/v) was dissolved in distilled water at 100°C, stirred using a magnetic stirrer (MS7‐H550‐S, Dlab, USA), and cooled to 45°C. Afterward, 1% (v/v) acetic acid and 0.25 mL glycerol per gram of CH were added as plasticizers and stirred for 15 min until fully dispersed. CEO coatings were prepared by adding 2% CEO and 0.5% (v/v) Tween 80 to distilled water and stirred for 45 min at room temperature (25°C ± 1°C) in a magnetic stirrer. AVG coatings were prepared by following the method of Navarro et al. (2011) with a concentration of 30% AVG. PR extract was prepared by grinding frozen PR into a fine powder, mixing 30% crude PR with 70% ethanol, and storing in dark glass vials with continuous shaking at 180 rpm for 3 days. The extract was filtered using Whatman paper No. 1 and stored at 4°C. The concentration obtained was considered 100%, and 10% CEO concentrations were prepared by diluting it with distilled water. The GA coating was prepared by dissolving 10% GA powder in distilled water, heating the solution to 40°C for 60 min, filtering it through muslin cloth, and incorporating 1% glycerol monostearate as a plasticizer. The pH was adjusted to 5.6 with 1 N NaOH.

For combined treatments (T7–T16), the respective coating solutions were mixed according to the specified concentrations, following the preparation process of individual components. The study was conducted using a completely randomized design (CRD) with three replications, each containing 20 fruits. A double‐coating protocol was employed for each treatment. The fruits were immersed in coating solutions for 3 min, then allowed to drain and air‐dry for 15 min under ambient conditions (∼20°C, > 75% RH) before a second coat was applied. The coated fruits were then stored at 20°C ± 1°C and 85%–90% relative humidity for 12 days. For evaluation, 4–5 fruits from each replication were sampled on days 03, 06, 09, and 12 after coating.

### 2.3 Quality Parameters of the Treated Guava

#### 2.3.1 Weight Loss

The weight loss of the guava samples was determined by comparing the initial weight with the weight after coating and expressing the results as a percentage. The weights were measured using an Entris BCE2201I‐1S balance (Sartorius AG, Germany) on Day 0 (initial weight) and on Days 3, 6, 9, and 12 (post‐coating weights). The percentage weight loss was calculated using the following formula, where W_1_ represents the initial weight and W2 represents the weight after coating.

$$\text{Weight loss}=\frac{w_{1}-w_{2}}{w_{1}}\times 100$$

#### 2.3.2 Firmness

The firmness of the guava was assessed using a penetrometer (GY‐2, Shijiazhuang Sanli Co. Ltd., China). A 3.5 mm diameter stainless steel probe was inserted into the fruit at three separate points per sample. The force applied during penetration was expressed in kg/cm^2^.

#### 2.3.3 Color

The color of the guava was measured using a Minolta CR‐410 Colorimeter (Konica Minolta Optics, Japan). The CIE color system was applied to assess the L* (lightness), a* (redness), and b* (yellowness) parameters. The hue angle (*h°*) was calculated using the following equation, where 0°, 90°, 180°, and 270° represent true red, yellow, deep green, and blue, respectively:

$$h^{{}^{\circ}}=\mathrm{ta}n^{-1}\frac{b^{*}}{a^{*}}\;\left(\text{For positive}\;b*\text{and positive}\;a*\text{value}\right)$$

$$h^{{}^{\circ}}=180^{{}^{\circ}}+\mathrm{ta}n^{-1}\frac{b^{*}}{a^{*}}\;\left(\text{For positive}\;b*\text{and negative}\;a*\text{value}\right)$$

#### 2.3.4 Total Soluble Solids

The total soluble solids (TSS) were determined using a digital refractometer (BOECO, Germany) and expressed as percent Brix, after applying a temperature correction at 20°C.

#### 2.3.5 pH

The pH of the guava was measured using a glass electrode pH meter (GLP 21, Crison, Barcelona, EEC), which was calibrated with pH 4.0 and pH 7.0 buffer solutions.

#### 2.3.6 Titratable Acidity

The titratable acidity (TA) was determined by following the method stated by Islam et al. (2013). In this procedure, 10 g of guava pulp was blended with 90 mL of distilled water and filtered through two layers of muslin cloth. Two to three drops of phenolphthalein were added to the filtrate as an indicator to determine the endpoint. The filtrate was then titrated with 0.1 N NaOH, and the results were expressed as apercentage.

#### 2.3.7 Total Sugar

The total sugar content of guava was measured using the method described by Islam et al. (2013) and expressed as apercentage.

#### 2.3.8 Ascorbic Acid

The ascorbic acid content of guava was determined using the method outlined by Ali et al. (2016). Briefly, 10 mL of juice was placed in a flask, and the volume was adjusted to 100 mL with 0.4% oxalic acid. A 5 mL aliquot was taken, and titration was carried out using 2,6‐dichloroindophenol. The ascorbic acid content was then calculated and expressed as mg/100 g.

#### 2.3.9 Total Phenolics

Total phenol content (TPC) was determined following the method described by Lin and Tang (2007), with absorbance measured at 760 nm. Phenol levels were quantified using a Gallic acid standard curve and expressed as mg GAE/100 g.

#### 2.3.10 Total Antioxidant

The total antioxidant concentrations were measured using the methods outlined by Krings and Berger (2001) and expressed as mM Trolox/100 g.

#### 2.3.11 Shelf Life Based on Marketability

The shelf life of guava fruit was determined by monitoring its marketability over time, focusing on appearance and spoilage. Daily evaluations were made to assess visible spoilage, texture, and overall condition. The shelf life was defined as the period (in days) until 50% of the sample became unmarketable. Once this occurred, the remaining fruits were discarded, and the elapsed time was recorded as the shelf life.

### 2.4 Statistical Analysis

The data were reported as mean ± standard deviation and analyzed using one‐way analysis of variance (ANOVA) in R software (Version 4.4.1). Mean separation was performed using Tukey's Honestly Significant Difference (HSD) test at a 5% level of significance (*p* ≤ 0.05). Further, Pearson correlation and principal component analysis (PCA) were analyzed to assess relationships and data patterns.

## 3 Results

### 3.1 Weight Loss, Firmness, and Color

**FIGURE 1:**
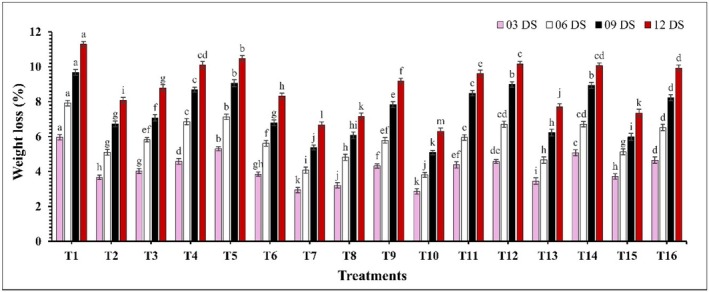
The impact of various edible coatings on weight loss of guava. All values are expressed as mean ± SD. DS = days of storage. Different lowercase letters indicate significant differences (*p* ≤ 0.05).

**FIGURE 2:**
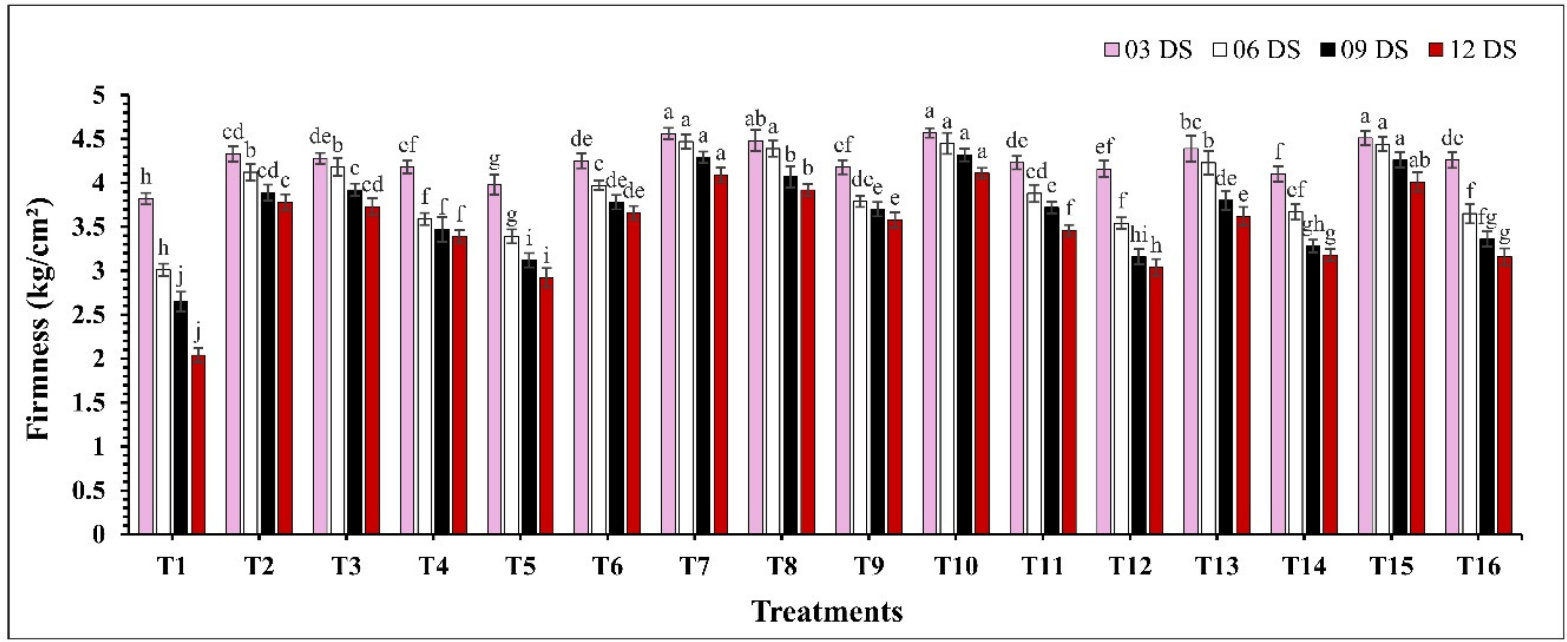
The impact of various edible coatings on the firmness of guava. All values are expressed as mean ± SD. DS = days of storage. Different lowercase letters indicate significant differences (*p* ≤ 0.05).

Significant differences (*p* ≤ 0.05) in weight loss percentages were observed across all analyzed samples during the storage period (Figure 1). At 12 days of storage (DS), control samples (T1) exhibited the highest weight loss at 11.29%, while the coated samples showed significantly lower weight loss (*p* ≤ 0.05). Moreover, treatment T10 (CH 2% + GA 10%) demonstrated the lowest weight loss at all storage intervals, with values of 2.86%, 3.81%, 5.09%, and 6.31% on 03, 06, 09, and 12 DS. Firmness decreased significantly (*p* ≤ 0.05) throughout the storage period, where coated samples maintained higher firmness compared to the control (Figure 2). At 12 DS, T10 (CH 2% + GA 10%) and T7 (CH 2% + CEO 2%) showed the highest firmness, with values of 4.11 kg/cm^2^ and 4.09 kg/cm^2^, while the control exhibited the lowest firmness at 2.04 kg/cm^2^. A significant change (*p* ≤ 0.05) in the surface color parameters of guava fruits under various treatments was observed and presented in Table 2. The L* significantly decreased (*p* ≤ 0.05) with the increasing storage period across all treatments. a* and b* both increased significantly (*p* ≤ 0.05) as the storage days advanced, with the rate of increase being notably slower in guavas subjected to coating treatments. Additionally, *h°* showed a significant (*p* ≤ 0.05) decline, with the most pronounced effect observed in the control sample. Among all treatments, T10 (CH 2% + GA 10%) again consistently maintained optimal color parameters throughout the storage period.

**TABLE 2:** The impact of various edible coatings on color attributes (lightness, a*, b*, and hue) of guava.

Treatments	03 DS	06 DS	09 DS	12 DS
L* (*lightness*)				
T1	56.34 ± 0.92^h^	42.12 ± 0.97^h^	37.56 ± 1.16^i^	33.92 ± 1.04^j^
T2	67.03 ± 0.87^d^	62.64 ± 0.72^cd^	58.32 ± 1.08^d^	54.58 ± 1.18^c^
T3	64.11 ± 0.71^e^	58.73 ± 0.65^ef^	52.98 ± 0.76^ef^	50.66 ± 0.57^e^
T4	61.51 ± 1.22^g^	58.33 ± 1.46^ef^	52.91 ± 0.93^ef^	48.76 ± 0.74^f^
T5	61.22 ± 0.84^g^	53.31 ± 1.22^g^	47.56 ± 0.81^h^	42.22 ± 0.76^i^
T6	66.89 ± 0.48^d^	61.32 ± 0.93^d^	56.89 ± 0.60^d^	53.02 ± 0.99^d^
T7	71.86 ± 1.38^a^	68.48 ± 0.79^a^	64.76 ± 1.02^b^	61.83 ± 0.75^a^
T8	71.27 ± 0.75^a^	67.96 ± 0.63^a^	65.34 ± 1.22^ab^	61.52 ± 0.84^a^
T9	64.01 ± 0.70^e^	59.08 ± 1.06^e^	53.11 ± 0.97^ef^	49.23 ± 0.96^ef^
T10	70.34 ± 1.02^ab^	69.02 ± 0.88^a^	66.57 ± 0.90^a^	62.76 ± 1.04^a^
T11	63.49 ± 1.23^ef^	57.13 ± 0.98^f^	49.72 ± 0.62^g^	44.16 ± 0.97^h^
T12	62.18 ± 1.19^fg^	59.26 ± 1.31^e^	51.57 ± 0.83^f^	47.16 ± 0.66^g^
T13	69.12 ± 0.97^bc^	65.33 ± 1.28^b^	60.87 ± 1.06^c^	58.97 ± 1.18^b^
T14	62.45 ± 0.84^ef^	54.21 ± 1.24^g^	48.36 ± 1.36^gh^	43.02 ± 0.77^hi^
T15	68.32 ± 0.90^cd^	64.21 ± 1.06^bc^	58.07 ± 0.79^d^	57.89 ± 0.65^b^
T16	63.01 ± 1.11^ef^	58.19 ± 0.73^ef^	53.39 ± 0.60^e^	49.13 ± 0.82^ef^
a* (*redness*)				
T1	−5.98 ± 0.29^a^	−1.31 ± 0.21^a^	1.63 ± 0.15^a^	2.79 ± 0.24^a^
T2	−9.31 ± 0.28^de^	−7.94 ± 0.13^f^	−6.87 ± 0.15^fg^	−6.04 ± 0.18^fg^
T3	−9.08 ± 0.19^d^	−7.23 ± 0.05^e^	−6.09 ± 0.27^e^	−5.68 ± 0.29^e^
T4	−8.33 ± 0.06^c^	−6.24 ± 0.13^c^	−5.48 ± 0.20^c^	−4.15 ± 0.13^c^
T5	−7.89 ± 0.17^b^	−5.87 ± 0.09^b^	−5.11 ± 0.09^b^	−3.43 ± 0.33^b^
T6	−9.12 ± 0.04^de^	−7.43 ± 0.19^e^	−6.68 ± 0.08^f^	−5.81 ± 0.14^ef^
T7	−10.02 ± 0.26^i^	−8.69 ± 0.10^hi^	−7.71 ± 0.14^hi^	−6.56 ± 0.20^hi^
T8	−9.92 ± 0.07^hi^	−8.71 ± 0.09^ij^	−7.87 ± 0.05^ij^	−6.87 ± 0.07^i^
T9	−9.38 ± 0.04^ef^	−8.43 ± 0.34^gh^	−7.54 ± 0.07^h^	−6.61 ± 0.24^hi^
T10	−9.88 ± 0.10^hi^	−8.96 ± 0.05^j^	−8.04 ± 0.06^j^	−6.84 ± 0.15^i^
T11	−8.28 ± 0.06^c^	−6.73 ± 0.28^d^	−4.99 ± 0.13^b^	−4.21 ± 0.27^c^
T12	−8.13 ± 0.05^bc^	−6.09 ± 0.09^bc^	−5.44 ± 0.09^c^	−4.02 ± 0.14^c^
T13	−9.56 ± 0.13^fg^	−8.65 ± 0.06^hi^	−7.01 ± 0.22^g^	−6.32 ± 0.11^gh^
T14	−8.08 ± 0.06^bc^	−6.79 ± 0.23^d^	−5.16 ± 0.04^b^	−3.62 ± 0.03^b^
T15	−9.72 ± 0.22^gh^	−8.35 ± 0.07^g^	−7.58 ± 0.07^h^	−6.69 ± 0.19^i^
T16	−8.26 ± 0.10^c^	−6.65 ± 0.06^d^	−5.74 ± 0.23^d^	−4.93 ± 0.07^d^
b* (*yellowness*)				
T1	38.89 ± 0.09^a^	45.03 ± 0.21^a^	49.11 ± 0.10^a^	51.55 ± 0.17^a^
T2	33.23 ± 0.18^g^	36.28 ± 0.19^i^	41.82 ± 0.07^e^	43.88 ± 0.10^g^
T3	34.67 ± 0.24^f^	38.53 ± 0.24^f^	43.72 ± 0.12^c^	46.65 ± 0.26^b^
T4	35.38 ± 0.10^d^	39.55 ± 0.08^d^	42.96 ± 0.05^d^	45.45 ± 0.09^d^
T5	36.12 ± 0.07^b^	42.05 ± 0.17^b^	44.76 ± 0.37^b^	46.48 ± 0.17^b^
T6	32.33 ± 0.25^h^	35.26 ± 0.34^j^	40.97 ± 0.27^g^	44.48 ± 0.12^f^
T7	31.24 ± 0.06^j^	33.23 ± 0.27^m^	35.67 ± 0.10^l^	41.34 ± 0.26^i^
T8	30.56 ± 0.29^k^	33.78 ± 0.09^l^	36.41 ± 0.16^k^	40.82 ± 0.23^j^
T9	33.18 ± 0.16^g^	37.24 ± 0.20^h^	41.46 ± 0.09^f^	43.25 ± 0.16^h^
T10	30.12 ± 0.06^l^	32.07 ± 0.17^n^	35.22 ± 0.07^m^	39.18 ± 0.19^k^
T11	34.89 ± 0.09^ef^	37.43 ± 0.15^h^	41.86 ± 0.12^e^	44.03 ± 0.13^g^
T12	35.17 ± 0.10^de^	39.14 ± 0.08^e^	41.21 ± 0.19^fg^	46.08 ± 0.26^c^
T13	32.52 ± 0.40^h^	34.76 ± 0.20^k^	40.23 ± 0.19^h^	43.89 ± 0.14^g^
T14	35.69 ± 0.10^c^	41.44 ± 0.39^c^	43.14 ± 0.12^d^	45.16 ± 0.09^d^
T15	31.86 ± 0.08^i^	33.33 ± 0.22^m^	37.16 ± 0.37^j^	41.02 ± 0.30^j^
T16	36.02 ± 0.18^b^	37.86 ± 0.12^g^	39.45 ± 0.20^i^	44.87 ± 0.06^e^
*h°* (*hue angle*)				
T1	98.75 ± 0.42^i^	91.66 ± 0.27^l^	88.10 ± 0.17^k^	86.91 ± 0.28^l^
T2	105.65 ± 0.51^d^	102.35 ± 0.13^e^	99.33 ± 0.18^f^	97.84 ± 0.21^e^
T3	104.68 ± 0.39^e^	100.62 ± 0.19^g^	97.93 ± 0.31^h^	96.94 ± 0.34^g^
T4	103.25 ± 0.06^f^	98.97 ± 0.17^ij^	97.27 ± 0.27^i^	95.22 ± 0.15^ij^
T5	102.32 ± 0.24^h^	97.95 ± 0.16^k^	96.51 ± 0.15^j^	94.22 ± 0.39^k^
T6	105.75 ± 0.17^d^	101.89 ± 0.20^f^	99.26 ± 0.06^f^	97.44 ± 0.20^f^
T7	107.78 ± 0.45^a^	104.65 ± 0.26^b^	102.20 ± 0.21^b^	99.02 ± 0.25^cd^
T8	107.99 ± 0.06^a^	104.47 ± 0.15^b^	102.20 ± 0.13^b^	99.55 ± 0.11^ab^
T9	105.78 ± 0.10^d^	102.75 ± 0.50^d^	100.31 ± 0.10^d^	98.68 ± 0.31^d^
T10	108.16 ± 0.15^a^	105.61 ± 0.06^a^	102.86 ± 0.13^a^	99.90 ± 0.18^a^
T11	103.35 ± 0.06^f^	100.19 ± 0.40^h^	96.80 ± 0.18^j^	95.46 ± 0.34^i^
T12	103.02 ± 0.08^fg^	98.85 ± 0.13^j^	97.52 ± 0.09^i^	94.98 ± 0.15^j^
T13	106.38 ± 0.26^c^	103.97 ± 0.09^c^	99.88 ± 0.29^e^	98.19 ± 0.18^e^
T14	102.75 ± 0.10^gh^	99.30 ± 0.23^i^	96.82 ± 0.08^j^	94.58 ± 0.13^k^
T15	106.97 ± 0.34^b^	104.07 ± 0.07^c^	101.52 ± 0.15^c^	99.26 ± 0.33^bc^
T16	102.92 ± 0.21^fg^	99.96 ± 0.12^h^	98.28 ± 0.36^g^	96.27 ± 0.09^h^

*Note:* Here, all values are expressed as mean ± SD. DS = Days of storage. Mean values with different superscript letters differ significantly (*p* ≤ 0.05) according to Tukey's HSD test.

### 3.2 Total Soluble Solids, pH, and Titratable Acidity

**FIGURE 3:**
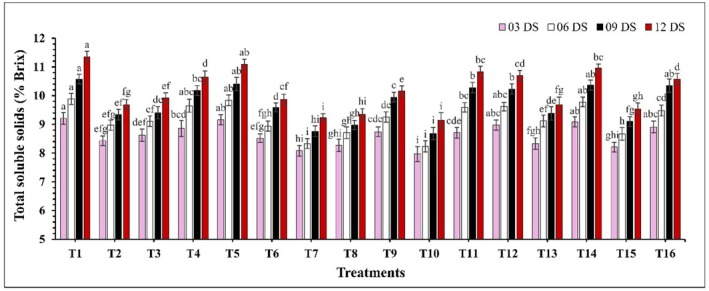
The impact of various edible coatings on total soluble solids of guava. All values are expressed as mean ± SD. DS = days of storage. Different lowercase letters indicate significant differences (*p* ≤ 0.05).

**FIGURE 4:**
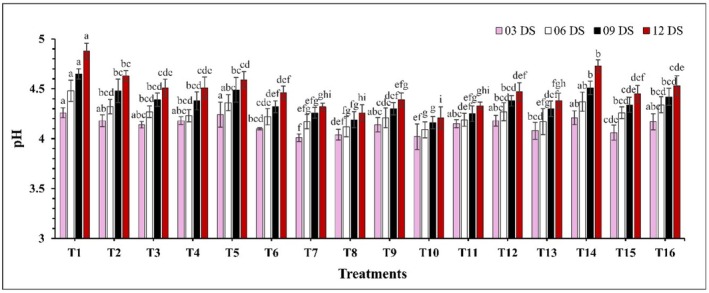
The impact of various edible coatings on pH of guava. All values are expressed as mean ± SD. DS = days of storage. Different lowercase letters indicate significant differences (*p* ≤ 0.05).

**FIGURE 5:**
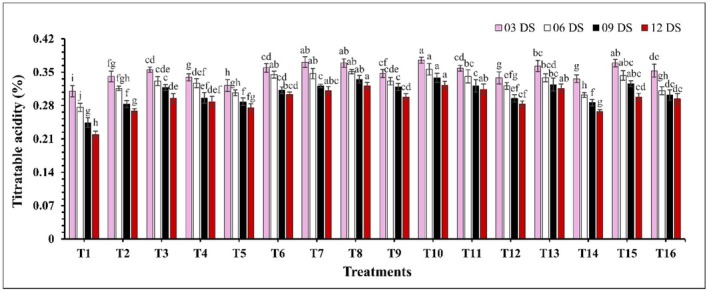
The impact of various edible coatings on titratable acidity of guava. All values are expressed as mean ± SD. DS = days of storage. Different lowercase letters indicate significant differences (*p* ≤ 0.05).

There was a significant difference (*p* ≤ 0.05) in TSS concentration (Figure 3). TSS levels increased with storage time, where uncoated guava showed a higher rate of increase compared to coated samples. At 12 DS, control samples exhibited the highest TSS concentration (11.36%), while the lowest concentrations were observed in treatments T10 (CH 2% + GA 10%) and T7 (CH 2% + CEO 2%) at 9.14% and 9.23%. Similarly, the pH of guava increased significantly (*p* ≤ 0.05) over the storage period (Figure 4). The largest fluctuations in pH were observed in the control and treatment T14, where pH values ranged from 4.26 to 4.88 and 4.21 to 4.73. In contrast, the smallest fluctuations in pH increase were observed in treatments T10 (CH 2% + GA 10%) and T8 (CH 2% + AVG 30%), where pH values ranged from 4.02 to 4.21 and 4.04 to 4.26. On the other hand, a significant decrease (*p* ≤ 0.05) in TA was observed over the storage period (Figure 5). Coated samples consistently exhibited higher TA levels compared to uncoated samples. At 12 DS, control samples showed the lowest TA percentage at 0.219%, while T10 (CH 2% + GA 10%) and T8 (CH 2% + AVG 30%) again displayed the highest TA levels at 0.322% and 0.321%.

### 3.3 Total Sugar, Ascorbic Acid, and Total Phenolics

**FIGURE 6:**
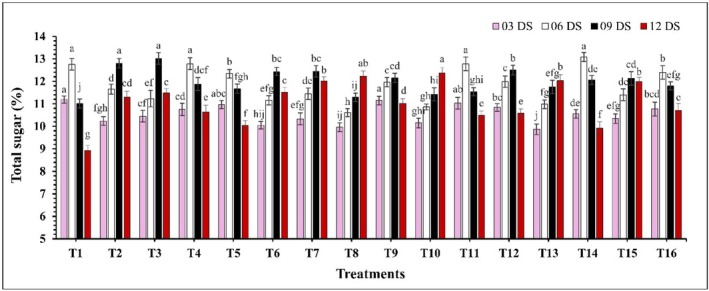
The impact of various edible coatings on total sugar of guava. All values are expressed as mean ± SD. DS = days of storage. Different lowercase letters indicate significant differences (*p* ≤ 0.05).

**FIGURE 7:**
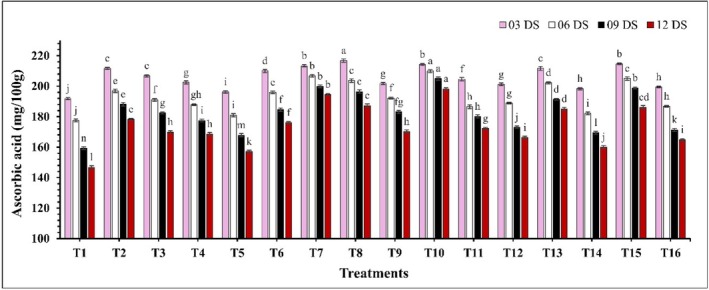
The impact of various edible coatings on ascorbic acid of guava. All values are expressed as mean ± SD. DS = days of storage. Different lowercase letters indicate significant differences (*p* ≤ 0.05).

**FIGURE 8:**
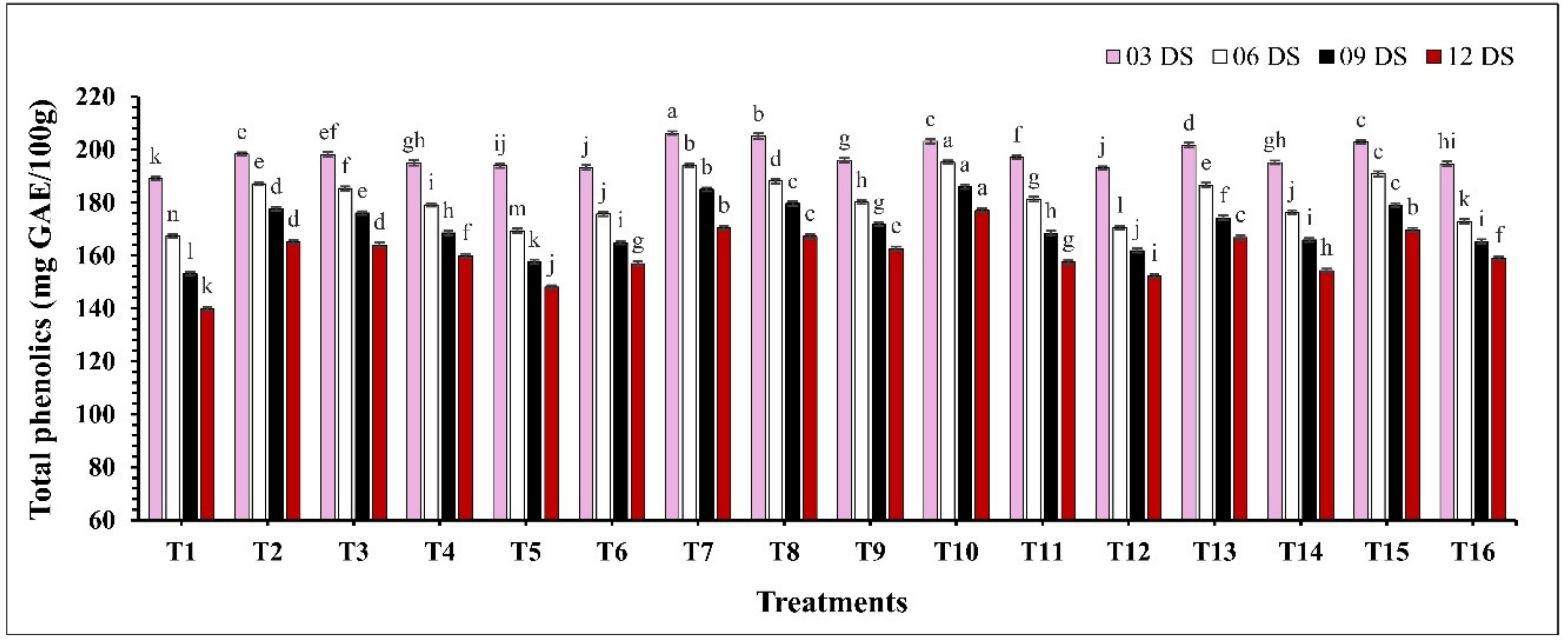
The impact of various edible coatings on total phenolics of guava. All values are expressed as mean ± SD. DS = days of storage. Different lowercase letters indicate significant differences (*p* ≤ 0.05).

A significant difference (*p* ≤ 0.05) in total sugar content was observed across all treatments during the storage period (Figure 6). Most of the treatments showed an initial increase in total sugar, followed by a decrease over the storage period. However, treatments T8 (CH 2% + AVG 30%), T10 (CH 2% + GA 10%), and T13 (CEO 2% + GA 10%) displayed a slow and consistent increase in total sugar throughout the measured period. Moreover, at 12 DS, the lowest total sugar percentage was recorded in control samples at 8.92%, while the highest percentage was found in treatment T10 (CH 2% + GA 10%) at 12.37%. In the case of ascorbic acid, a significant decline (*p* ≤ 0.05) in ascorbic acid levels was noted with increased storage period (Figure 7). This decline was more pronounced in uncoated samples compared to coated samples. Among all treatments, T10 (CH 2% + GA 10%) maintained the highest ascorbic acid content (214.21–198.22 mg/100 g) throughout the storage period, while the control (T1) exhibited the lowest (191.88–146.74 mg/100 g) ascorbic acid levels. Furthermore, a significant effect (*p* ≤ 0.05) of the edible coating on the TPC was observed. Over the storage period, a marked decline in TPC was noted (Figure 8). The highest TPC was again found in treatment T10 (CH 2% + GA 10%), with values ranging from 203.13 to 177.03 mg GAE/100 g. In contrast, the control samples exhibited the lowest phenolic content, which decreased from 189.12 to 139.82 mg GAE/100 g.

### 3.4 Total Antioxidant and Shelf Life Based on Marketability

**FIGURE 9:**
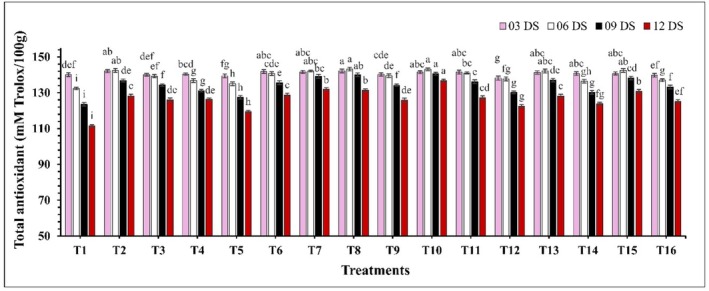
The impact of various edible coatings on total antioxidants of guava. All values are expressed as mean ± SD. DS = days of storage. Different lowercase letters indicate significant differences (*p* ≤ 0.05).

**FIGURE 10:**
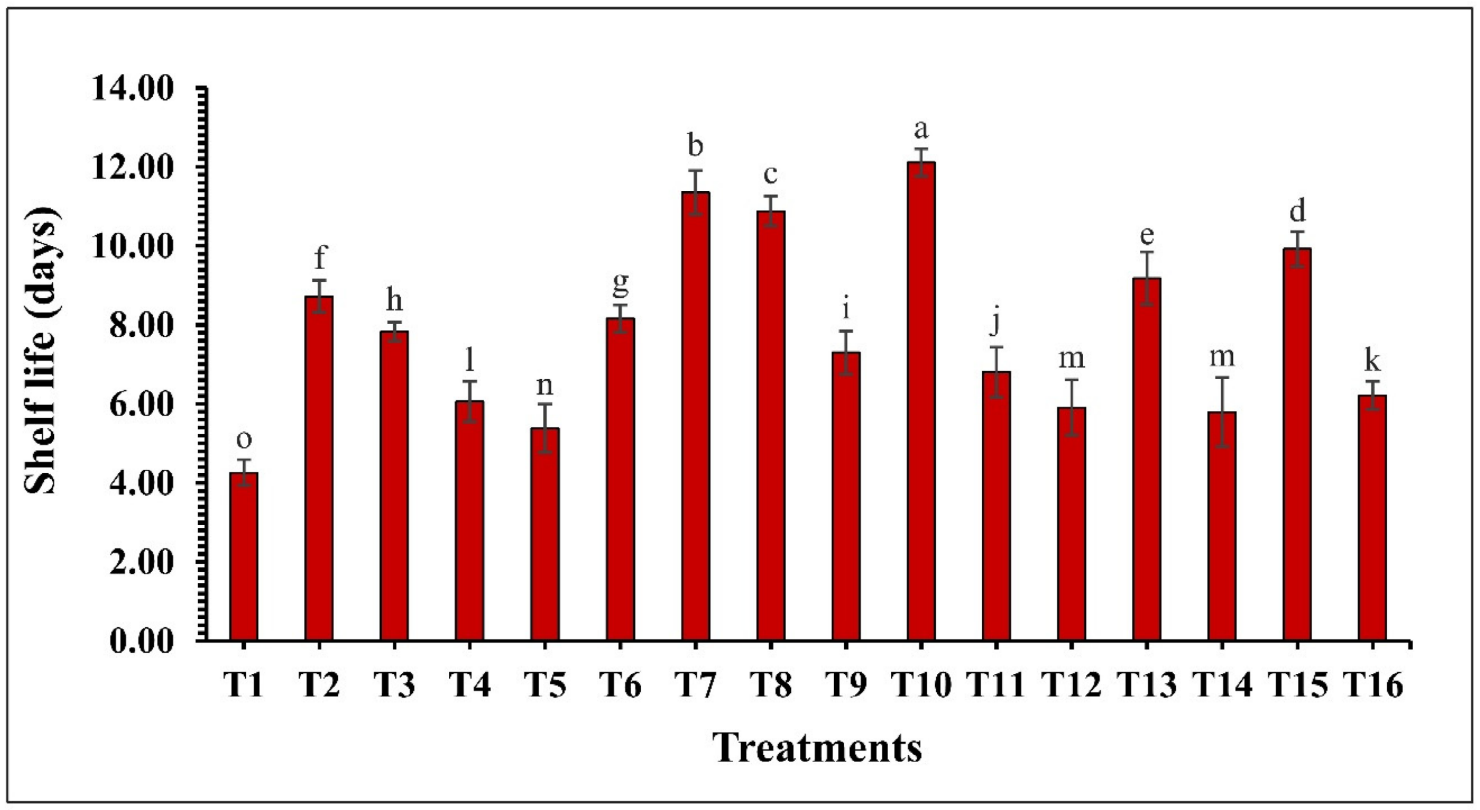
The impact of various edible coatings on shelf life of guava. All values are expressed as mean ± SD. DS = days of storage. Different lowercase letters indicate significant differences (*p* ≤ 0.05).

The effect of edible coatings on the total antioxidant content was significant (*p* ≤ 0.05) throughout the storage period. Coated treatments exhibited an increasing trend in antioxidant levels up to 6 DS, while the control (T1) showed a declining trend (Figure 9). Notably, the control had a more substantial decrease in antioxidant content compared to the coated treatments. Among the coated treatments, T10 (CH 2% + GA 10%) maintained the highest antioxidant content, ranging from 141.52 to 136.76 mM Trolox/100 g, whereas the control group (T1) recorded the lowest values, ranging from 140.08 to 111.55 mM Trolox/100 g. Moreover, shelf life based on marketability of guava was also significantly influenced (*p* ≤ 0.05) by the different treatments (Figure 10). Among the treatments, the control group (T1) exhibited the shortest shelf life, with an average of 4.25 days. In contrast, all treated fruits significantly extended the shelf life, with T10 (CH 2% + GA 10%) again demonstrating the best performance with an extended marketable period up to 12 days.

### 3.5 Correlation of Physicochemical Parameters in Guava

**FIGURE 11:**
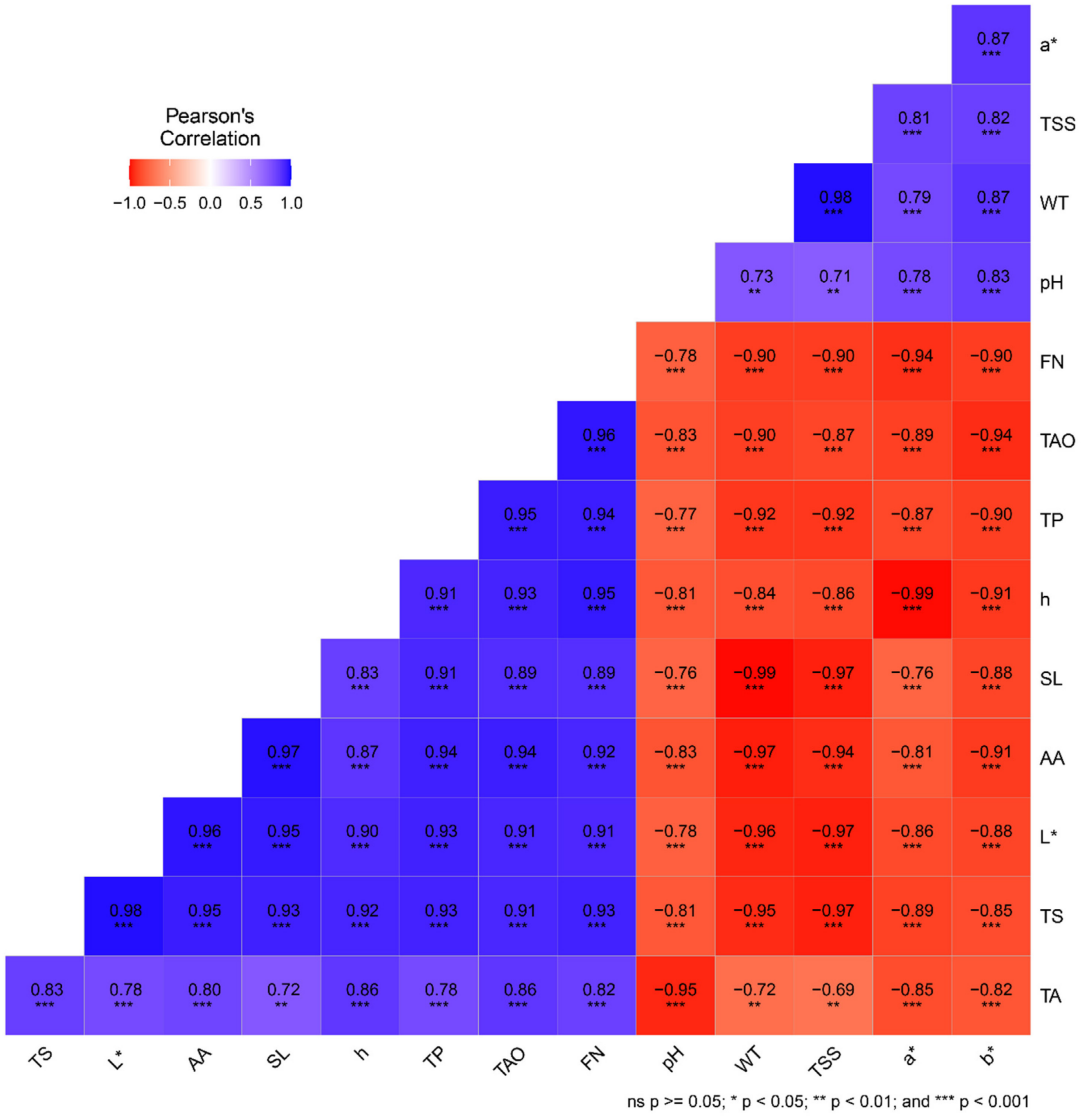
Pearson (*n*) correlation between various parameters studied in guava fruits. a*, *redness*; AA, ascorbic acid; b*, *yellowness*; FN, firmness; h, hue angle; L*, lightness; SL, shelf life; TAO, total antioxidants; TA, titratable acidity; TP, total phenolics; TSS, total soluble solids; TS, total sugar; WT, weight loss.

**FIGURE 12:**
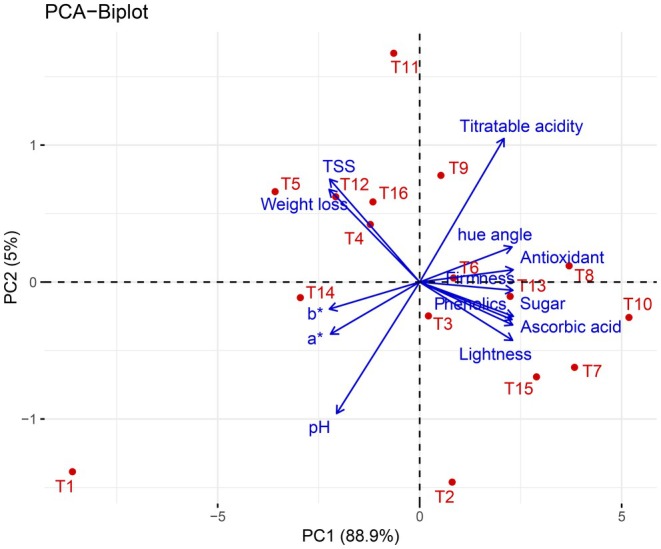
Principal component analysis (PCA) of the different treatments across various physicochemical parameters.

Pearson (*n*) correlation exhibited significant relationships among various physicochemical parameters in guava (Figure 11). A strong positive correlation (*p* < 0.001 and *p* < 0.01) was observed between total sugar, L*, ascorbic acid, shelf life, *h°*, TPC, total antioxidants, firmness, and TA. Additionally, a*, b*, TSS, weight loss, and pH also exhibited significant positive associations (*p* < 0.001 and *p* < 0.01) with these parameters. Conversely, a strong negative correlation (*p* < 0.001 and *p* < 0.01) was found between pH, weight loss, TSS, a*, b*, TA, total sugar, L*, ascorbic acid, shelf life, *h°*, TPC, total antioxidants, and firmness. Further PCA was performed to assess the relationships among various parameters across different treatments and their impact on guava quality during storage (Figure 12). The analysis indicated that the first principal component (PC1) explained 88.9% of the total variance, while the second principal component (PC2) accounted for 5%. PC1 showed a strong positive correlation with weight loss, TSS, TA, *h°*, and total antioxidant. In contrast, PC2 exhibited a positive association with firmness, total sugar, ascorbic acid, TPC, and L*. The spatial clustering of the treatments revealed distinct groupings based on these properties, emphasizing the significant influence of the applied treatments on guava quality throughout the storage period.

## 4 Discussion

Physiological weight loss is a key factor affecting the shelf life and quality attributes of guava (Mahin et al. 2025). This weight loss is primarily caused by natural enzymatic processes and the differences in water vapor pressure between the internal and external environments, which lead to moisture loss (Gidado et al. 2024; Sahoo et al. 2015). As the storage period progresses, increased respiration, transpiration, ethylene production, and cellular disruptions contribute to the deterioration of guavas, resulting in weight loss and reduced market value (Bashir and Abu‐Goukh 2003; Gill et al. 2016). The present study demonstrated that control samples experienced the highest weight loss, while guavas treated with a combination of CH 2% and GA 10% exhibited significantly lower weight loss, indicating the protective effects of edible coatings. By increasing carbon dioxide levels inside the fruit and limiting oxygen availability, coatings inhibit respiratory enzymes and consequently reduce respiration and transpiration rates (Priya et al. 2023). Additionally, coatings such as CH and GA act as physical barriers to moisture loss, delaying dehydration and preventing fruit shriveling (Hong et al. 2012; El‐Gioushy et al. 2022).

Firmness, which is another crucial factor for determining customer acceptability, is also impacted by weight loss. The softening of guava fruit is often linked to ethylene activity, which accelerates at room temperature, peaking within 4 days after harvest (Paul et al. 2023; Dutta Roy et al. 2023). This softening is associated with the metabolism of cell wall carbohydrates, where hydrolytic enzymes promote pectin solubilization, destabilizing the primary cell wall and middle lamella, thus weakening the fruit's structure (Chen et al. 2015). In the present study, the control samples exhibited the lowest firmness, while the highest firmness was recorded in guavas treated with the combination of CH 2% and GA 10%.

As a climacteric fruit, guava continues to respire after harvest, with respiration and ethylene production peaking within 2–3 days at ambient temperatures (Yousaf et al. 2024). Changes in fruit color, particularly in hue (h*) and lightness (L*), are essential markers of ripening. As guava ripens, chlorophyll degradation leads to the yellowing of the fruit, marked by changes in the color coordinates, especially increases in the “a*” (redness) and “b*” (yellowness) values (Forato et al. 2015). In this study, control samples exhibited rapid color change from green to yellow by day 6, indicating chlorophyll degradation or enzymatic reactions like the Maillard process (Aguiló‐Aguayo et al. 2009). On the other hand, coated guavas, particularly those treated with CH 2% + GA 10%, retained a greener color, likely due to the reduced respiration rate and suppressed ethylene production, which slowed the ripening process (Nur Hanani et al. 2023). Previous studies also demonstrate that CH and GA coatings effectively preserved the color of both guavas and sweet peppers (Hong et al. 2012; Xing et al. 2011). Moreover, hue angle (h*) analysis confirmed these results, showing a rapid decline in the control samples, with values shifting from the green quadrant (180°) to the yellow quadrant (90°) (Mclellan et al. 1995). In contrast, the guavas coated with CH 2% + GA 10% maintained a higher h* value, indicating delayed ripening and reduced browning.

The quality attributes of guava, such as TSS, pH, TA, and total sugar content, are essential indicators of fruit maturity and ripening during storage. This study demonstrated that with increasing storage periods, TSS, pH, and total sugar content typically increase, while TA decreases—a common phenomenon observed in climacteric fruits (Gill et al. 2016). These changes are primarily driven by metabolic and biochemical processes that occur during ripening (Wang et al. 2024). The increase in TSS is closely linked to the breakdown of starch into soluble sugars, playing a crucial role in the development of sweetness and flavor (Durán‐Soria et al. 2020). The results indicated a consistent increase in TSS and total sugar content in control, while guavas treated with CH 2% + GA 10% coating exhibited a slower rate of increase. This suggests that the coating effectively delayed ripening by minimizing starch hydrolysis. Similarly, pH increases during fruit maturation as organic acids are metabolized and converted into sugars (Li et al. 2020). The results showed that control samples had the highest pH, while the CH 2% + GA 10% treated samples maintained a lower pH. A lower pH creates unfavorable conditions for microbial growth, indicating that the CH 2% + GA 10% coating may help control microbial activity (Factors Affecting Microbial Growth in Foods 2016). TA, another critical quality factor, typically decreases during ripening as organic acids are consumed in respiratory processes (Anthon et al. 2011). Results demonstrated that the application of the CH 2% + GA 10% coating helps to preserve the higher levels of TA by slowing the conversion of organic acids into sugars. This effect is consistent with previous research on coated fruits, such as mangoes and bell peppers, where coatings delayed increases in TSS, pH, total sugar, and the reduction of TA (Kumar et al. 2023; Ullah et al. 2017).

As previously discussed, guava fruit is often considered a “super fruit” due to its rich content of bioactive compounds, particularly ascorbic acid, phenolics, and antioxidants, which contribute to its health‐promoting properties. However, the stability of these compounds during storage is a concern, as they are known to degrade over time. In this study, results demonstrated a significant reduction in the levels of ascorbic acid, phenolic compounds, and total antioxidants in guava during storage, with the most significant reductions observed in uncoated guava fruits. Ascorbic acid, a key antioxidant compound with nonenzymatic properties, is particularly susceptible to degradation through autoxidation during storage (Zaidi et al. 2023). In the present study, uncoated guavas exhibited a rapid decrease in ascorbic acid content over time, whereas guavas coated with CH 2% + GA 10% showed significantly reduced degradation. This may be attributed to the coating's ability to limit oxygen uptake, thereby reducing oxidative degradation of ascorbic acid and helping to preserve its concentration throughout the storage period (Maqbool et al. 2011; Pham et al. 2023).

In addition to ascorbic acid, phenolic compounds are crucial antioxidants that serve as protective mechanisms in fruits (Maqbool et al. 2011). The presence of phenols is crucial as they help scavenge reactive oxygen species (ROS), preventing lipid peroxidation and oxidative damage in plant tissues (El‐Gioushy et al. 2022; Lo'ay and Doaa 2020). Results demonstrated that the CH 2% + GA 10% coating helps maintain higher levels of total phenolic content and total antioxidants in guavas, thereby enhancing their oxidative stability during storage. These findings are consistent with previous studies indicating that edible coatings can significantly reduce ROS accumulation, thereby protecting the bioactive compounds in fruit. Moreover, the preserved levels of physicochemical properties contributed to an extended shelf life of the fruit. These results align with earlier studies that highlighted the benefits of edible coatings in prolonging the storage life of fruits (Blancas‐Benitez et al. 2022; Pham et al. 2023; Priya et al. 2023).

## 5 Conclusion

This study demonstrates that edible coatings significantly enhance the postharvest quality and extend the shelf life of guava fruits by mitigating physiological and biochemical deterioration. Coated samples exhibited reduced weight loss, maintained higher firmness, and showed slower changes in color, TSS, pH, and total sugar compared to the control. Furthermore, the coating effectively preserved TA, ascorbic acid, phenolic content, and total antioxidant, contributing to the extended shelf life and retention of nutritional value. Among the formulations tested, the combination of CH 2% and GA 10% emerged as the most effective, indicating strong synergistic potential for maintaining fruit integrity and prolonging storability. These findings highlight the potential of CH 2% + GA 10% coatings as a sustainable, non‐toxic, and efficient postharvest strategy for guava. Future studies should investigate the incorporation of different storage conditions and packaging materials with this coating solution to fully assess its potential.

## Author Contributions

**Litun Ahmed Labib:** conceptualization (lead), data curation (lead), formal analysis (lead), investigation (lead), methodology (lead), supervision (supporting), validation (lead), visualization (lead), writing – original draft (lead), writing – review and editing (lead). **Swagata Ahmed:** conceptualization (equal), data curation (equal), formal analysis (equal), investigation (equal), methodology (equal), validation (equal), visualization (equal), writing – original draft (equal), writing – review and editing (equal). **Md. Fakhrul Hasan:** conceptualization (equal), methodology (equal), resources (lead), supervision (lead), validation (lead), writing – review and editing (supporting).

## Ethics Statement

The authors have nothing to report.

## Conflicts of Interest

The authors declare no conflicts of interest.

## Data Availability

All data supporting the reported results are included in the article in the form of tables and figures. Raw data will be made available on request.
